# Dynamic response characteristics and damage rule of graphite ore rock under different strain rates

**DOI:** 10.1038/s41598-023-28947-9

**Published:** 2023-02-07

**Authors:** Haiwang Ye, Xingwang Li, Tao Lei, Lifeng Li, Qizhou Wang, Ning Li

**Affiliations:** 1 School of Resources and Environment Engineering, Wuhan University of Technology, Wuhan, 430070 Hubei China; 2 Hubei Key Laboratory of Mineral Resources Processing and Environment, Wuhan University of Technology, Wuhan, 430070 Hubei China

**Keywords:** Engineering, Civil engineering

## Abstract

In the process of mining graphite mine, rock mass is often subjected to dynamic loads such as blasting or mechanical crushing, which involves dynamic responses of different strain rates, and blasting and crushing effect are affected by the rock dynamic properties and damage specials. The dynamic response characteristics and damage rule of graphite ore rock under different strain rates are very important but rarely studied in the past. To study these issues and provide support for graphite ore rock mining, the dynamic compression tests of graphite ore rock under five kinds of impact pressures were designed and carried out by using the Split Hopkinson Pressure Bar (SHPB) test system, combining with the high-speed photography system and crushing screening tests. The dynamic characteristics, crushing process, crushing mode, crushing form and fragmentation distribution of the graphite ore rock under different strain rates were analyzed. The results show that the dynamic characteristics of the graphite ore rock have obvious strain rate effect. The hardening coefficient (*DIF*) is positively correlated with the cubic root of strain rate, and the softening factor (*K*) is negatively correlated with the cubic root of strain rate. Shear failure mainly occurs in the graphite ore rock under impact load, and the crushing process can be divided into five stages, they are compaction, crack initiation, crack expansion and penetration, fragmentation collision and fragmentation fall. In addition, the crushed blocks are mainly triangular pyramid (or cone-like) fine granular and powder. The broken fragments of the graphite ore rock are in accord with the fractal geometry characteristics. That is, the average broken particle size (*d*_*S*_) decreases linearly with the increase of strain rate, and the fractal dimension (*D*_*a*_) increases weakly with the increase of strain rate. Based on D-P fracture criterion and Weibull distribution model, the dynamic damage constitutive model of the graphite ore rock was established, and the correlation between strain rate and Weibull distribution parameters (*m* and *F*_*0*_) was used to reasonably modify the damage constitutive model. The modified damage constitutive model curve is in good agreement with the experimental curve, which can basically reflect the strain rate effect of the dynamic characteristics of the graphite ore rock and the evolution characteristics of the dynamic stress–strain curve at different stage.

## Introduction

In recent years, with the rise of new energy and new material industry, graphite is gradually becoming an irreplaceable and important raw material in the fields of national defense, aerospace, and new materials^1^. Both at home and abroad, the exploitation of graphite resources is continuously increasing, and to clear the rock mechanics properties of graphite mine is becoming more and more important. Accordingly, how to exploit graphite resources safely, economically and efficiently is an important issue that we have to focus on. As we all know, in the process of mining, including drilling, blasting, mechanical crushing, etc., the rock will be subjected to dynamic loads in different degrees^2^. Under these dynamic loads, the strain rate of rock ranges from 10^1^ to 10^3^ s^−1^, and sometimes the strain rate caused by a blast may even reach 10^4^ s^−1^^3,4^. Within these strain rate ranges, rock will show different mechanical response characteristics and damage rule from those under static loading. In this case, it is obviously inappropriate to study the dynamic properties of rock by using related theories of statics^5–7^. Therefore, in order to provide theoretical basis for graphite mine to realize the high efficiency of ore body mining and economic crushing process, it is necessary to conduct in-depth research on dynamic response characteristics and damage rule of graphite ore under different strain rates.

As the main tool of dynamic performance test of rock materials, the Split Hopkinson Pressure Bar (SHPB) system can not only test the relationship between the stress, strain and other mechanical parameters of rock specimens and strain rates, but also obtain the rock breakage characteristics under different strain rates, which provides important reference materials for engineering practice. So SHPB system has been widely concerned by researchers from various countries^8^. Many researchers have studied the dynamic properties of various rocks using the SHPB test system. In the study of rock dynamic characteristics, as early as 1968, Kumar^9^ introduced SHPB test technology into the test of rock dynamic strength, and studied the influence of stress rate on the strength of basalt and granite. Subsequently, some researchers tried to use the SHPB system to test the dynamic strength of rocks^10–15^. And they all founded that the dynamic strength of rock increases with the increase of strain rate. Based on abundant test data, Li et al.^16^ concluded that the relationship between rock crushing strength and strain rate is $\sigma_{d} = A\dot{\varepsilon }^{B}$, where *B* is about 0.3, while the value of *A* varies with different rock. This important conclusion has been widely recognized by scholars in the field of rock dynamics. With the gradual maturity of SHPB test technology, more and more dynamic characteristics tests of different rock have been carried out. These studies finished by^17–28^ respectively show that the dynamic strength of rock has obvious strain rate effect, which is quite consistent with the conclusion of Li et al.^16^. However, at present, there is no very clear conclusion about the strain rate effect on dynamic peak strain and elastic modulus parameters of rock, which may be where future research needs to break through.

One significant issue noted from the literature is that the reported SHPB tests are also focus on the crushing characteristics and the damage constitutive model of rock. In terms of the study of dynamic crushing characteristics of rock, magnetite impact experiments under different strain rates have been carried out by Li et al.^26^, who revealed the distribution law of the lumpiness of magnetite under dynamic load, and obtained the reasonable range of strain rates to realize the crushing of magnetite. Huang et al.^29^ carried out the SHPB test of frozen cement solidified soil, and the influence of impact velocity on the viscoplastic failure characteristics of frozen cement soil was analyzed. Wang et al.^30^ carried out a study on the fractal characteristics of the pomegranate biotite schist under impact loading, which provided a great reference for the analysis of dynamic crushing mechanism, crushing block size distribution, and crushing energy consumption of the roadway surrounding rock. In the research of rock dynamic damage constitutive model, Zheng et al.^31^ established a coal rock strength-type statistical damage constitutive model based on dynamic mechanical properties. Through the combined model method, a dynamic constitutive model of sandstone damage was constructed by Jiang et al.^32^, which accurately described the dynamic mechanical properties of sandstone under impact. Hao et al.^33^ combined the continuous damage theory with statistical strength theory, and established the strength constitutive model of magnetite under dynamic load. Zhang et al.^34^ carried out an investigation on damage characteristic and constitutive model of deep sandstone under coupled high temperature and impact loads. Because these dynamic constitutive models based on statistical strength are not difficult to understand theoretically and require fewer solving parameters, it is more concerned by researchers and easier to apply to engineering practice.

The above research results indicate that with the development of modern geotechnical engineering and the maturation of SHPB test system, the study of rock dynamic properties has been more in-depth and extensive. However, most researches on rock dynamic characteristics focus on the pre-peak mechanical properties such as peak strength, peak strain and dynamic elastic modulus, while few studies on the post-peak mechanical properties. In addition, studies on dynamic fracture characteristics of rock mainly focus on the relationship between fragmentation distribution and energy consumption characteristics, and fewer studies are related to strain rates. The post-peak mechanical properties of rock can reflect the residual bearing capacity and anti-deformation limit of rock in the later stage of failure under load, which can provide key information for the study of many engineering hazards such as rockburst and mass collapse^35^. Considering the crushing characteristics of rock from the view of strain rate effect, the reasonable strain rate range of broken rock can be obtained, which can provide important basis for blasting mining and rock mechanical crushing. What’s more, there are obvious differences in mineral composition and structural characteristics of different rocks, and their damage rules and constitutive relations must also be different. Consequently, it is still of great significance to carry out studies on dynamic response characteristics and damage rules of graphite ore rock under different strain rates.

Relying on the open-pit mining project of a graphite mine in Luobei, Heilongjiang Province, China, high grade crystalline graphite ore rocks with high value were selected as the test materials to carry out SHPB impact compression tests under different strain rates. The stress–strain curves of the whole dynamic crushing process were obtained, and the strain rate effect of pre-peak and post-peak dynamic characteristics was analyzed. At the same time, the dynamic crushing characteristics of graphite ore rock were accurately described by high-speed photography system and crushing body screening test. Based on the statistical analysis results, the distribution law of graphite ore rock crushing lumpiness under different strain rates was explored. Furthermore, combined with D-P fracture criterion and Weibull distribution model, the dynamic damage constitutive model of graphite ore rock was established, and the damage constitutive model was modified by using the correlation between strain rate and Weibull distribution parameters (*m* and *F*_*0*_). It is expected that the research can reveal the dynamic response characteristics and damage rule of graphite ore rock, so as to facilitate the efficient and reasonable mining of graphite mine.

## Experiments

### Sample preparation and basic mechanical properties

**Figure 1:**
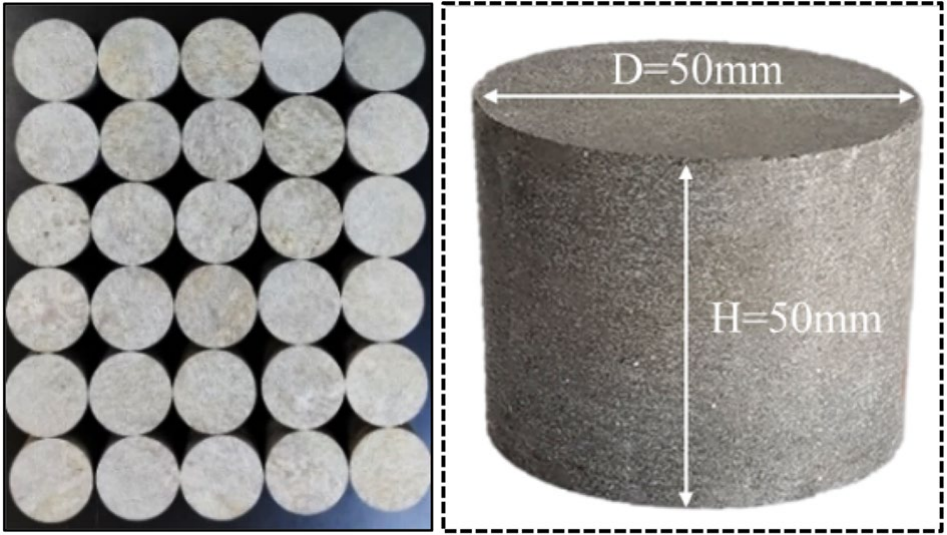
Graphite ore rock samples.

The rock material used in this test is from a graphite mine in Luobei, Heilongjiang province, China, and the sample rock is high grade crystalline graphite ore. The raw graphite ore rock was obtained from the mine site, and the samples were processed through the procedures of drilling, cutting, face grinding and leveling measurement. In order to reduce the influence of discreteness and anisotropy on rock mechanical properties, the test samples were taken from the same raw rock. According to the *Engineering Rock Mass Test Method Standard* (GBT50266-2013)^19^ and ISRM^2^, the test samples were processed into cylinders with a diameter of 50 mm and a height of 50 mm. The test samples are shown in Fig. 1, and the basic mechanical parameters of the ore rock samples are listed in Table 1.

**Table 1:** Basic mechanical parameters of the tested graphite ore samples.

*σ*_*c*_/MPa	*E*/GPa	*μ*	*C*/MPa	*φ/*^***°***^
78.60	45.76	0.26	20.21	29.50

In Table 1, *σ*_*c*_ is uniaxial compressive strength, *E* is modulus of elasticity, *μ* is Poisson Ratio, *C* is cohesive force, *φ* is angle of internal friction.

### Test equipment and principle

**Figure 2:**
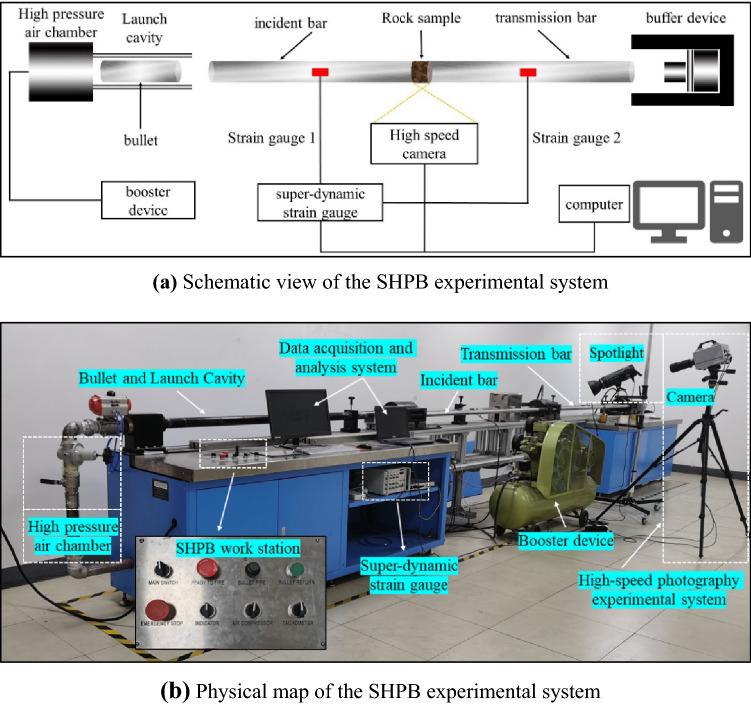
SHPB experimental system used in this paper. (**a**) Schematic view of the SHPB experimental system; (**b**) Physical map of the SHPB experimental system.

The SHPB system adopted in this test is shown in Fig. 2, which is mainly composed of a booster device, a high-pressure air chamber, a launch cavity, a bullet, an incident bar, a transmission bar, a super-dynamic strain gauge, a buffer device, a high speed camera and a computer (data acquisition and analysis system). The incident bar and the transmission bar are made of 18Ni, whose elastic modulus and longitudinal wave velocity are 190 GPa and 4900 m s^−1^, respectively. Both the incident bar and the transmission bar have a length of 2000 mm and a diameter of 50 mm. The bullet is 400 mm long and 50 mm in diameter.

According to the homogenization conditions of the SHPB system and the one-dimensional elastic stress wave theory, the three-wave method was used to process the test data. The waveform signals measured and recorded by strain gauge and ultra-dynamic strain gauge were statistically analyzed and calculated, and the dynamic parameters of the samples, including stress $\sigma$, strain $\varepsilon$ and strain rate $\dot{\varepsilon }$ were obtained. The principle of three-wave method^36^ can be expressed by Eqs. (1)–(3).

1 $$\sigma \left( t \right) = \frac{{A_{e} }}{{2A_{s} }}\left[ {\sigma_{I} \left( t \right) - \sigma_{R} \left( t \right) + \sigma_{T} \left( t \right)} \right]$$

2 $$\varepsilon \left( t \right) = \frac{1}{{\rho_{e} C_{e} L_{s} }}\mathop \smallint \nolimits_{0}^{t} \left[ {\sigma_{I} \left( t \right) + \sigma_{R} \left( t \right) - \sigma_{T} \left( t \right)} \right]dt$$

3 $$\dot{\varepsilon }\left( t \right) = \frac{1}{{\rho_{e} C_{e} L_{s} }}\left[ {\sigma_{I} \left( t \right) + \sigma_{R} \left( t \right) - \sigma_{T} \left( t \right)} \right]$$

where $\sigma_{I} \left( t \right)$,$\sigma_{R} \left( t \right)$,${ }\sigma_{T} \left( t \right)$ are the incident stress, reflection stress and transmission stress corresponding to *t* at a certain time respectively; $\rho_{e} C_{e}$ is the wave impedance of the elastic rod; $L_{s}$ is the length of sample; $A_{e}$ and $A_{s}$ are the cross sectional areas of the elastic rod and the sample respectively.

**Figure 3:**
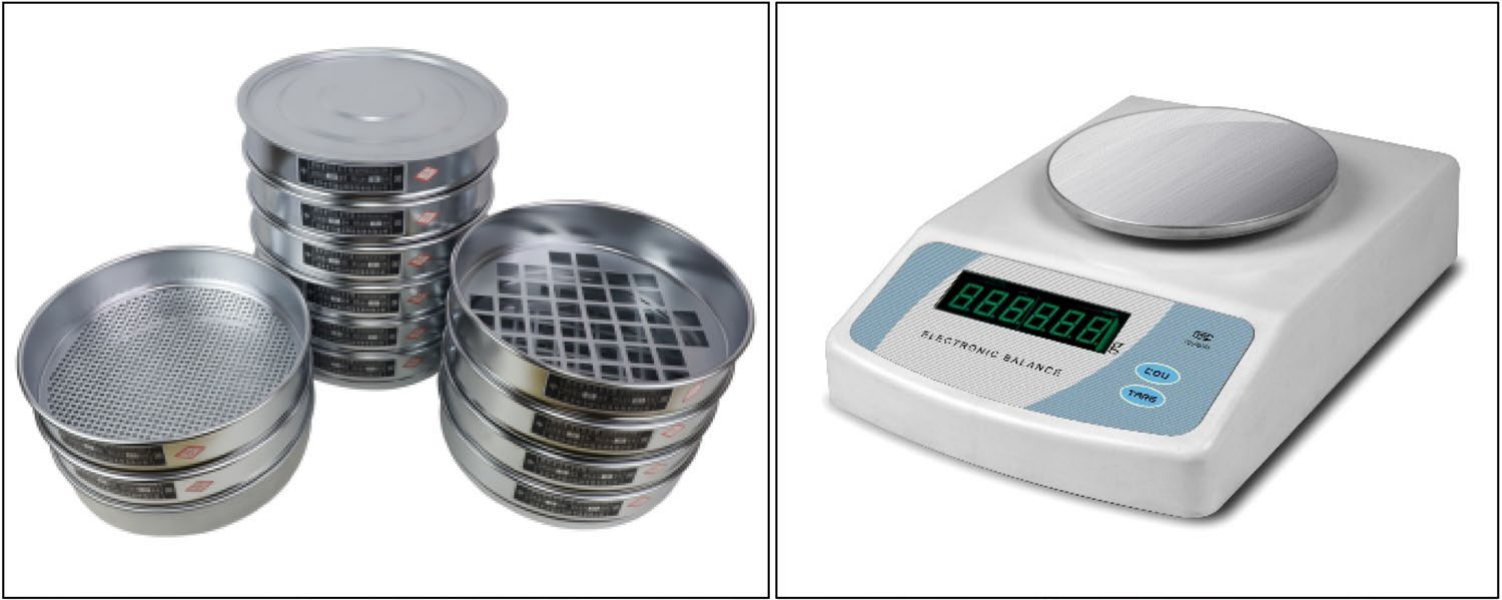
Square hole sieve and high-precision electronic balance.

After the sample crushing is completed, the square hole sieve with eight sizes of 40–50 mm, 31.5–40 mm, 20–31.5 mm, 16–20 mm, 10–16 mm, 5–10 mm, 2.5–5 mm and < 2.5 mm were used respectively to screen the fragments of the broken samples. A high-precision electronic balance was used to weigh and record the mass of fragments of each size grade after screening. The square hole sieve and high-precision electronic balance adopted in this test are shown in Fig. 3.

### Test process and results

Before the test, in order to reduce the influence of end face friction effect on the test results, butter was evenly spread on the contact end of the sample and the rod, and the sample and the rod were in close contact. Then the bullet was returned to the bottom of the air chamber to ensure that the bullet was shot from the same position in each test. After that, the impact pressure was adjusted successively to 0.2 MPa, 0.3 MPa, 0.4 MPa, 0.5 MPa and 0.6 MPa to complete the impact loading test. In order to reduce the discreteness of the data, five samples were prepared for each group of impact pressure, and three samples with low dispersion were selected from each group to analyze the test results. Dynamic parameters of the graphite ore rock samples obtained by the impact tests are listed in Table 2.

**Table 2:** Dynamic parameters of ore rock samples.

*P*/MPa	The sample number	$\dot{\varepsilon }$/s^−1^	$\sigma_{d}$/MPa	*E*_*d*_*/*GPa	$\varepsilon_{d}$	$\varepsilon_{0}$	$\varepsilon_{max}$	*DIF*	*K*
0.2	5-10-3	103.007	106.879	36.021	0.005019	0.004080	0.01996	1.360	0.204
	5-10-4	95.129	98.463	36.695	0.004935	0.002742	0.01441	1.253	0.190
	5-10-5	110.328	109.300	35.136	0.005425	0.004825	0.02377	1.391	0.203
0.3	5-10-7	167.851	136.903	31.184	0.007140	0.005917	0.03434	1.742	0.172
	5-10-8	161.163	135.419	32.341	0.006616	0.005440	0.03318	1.723	0.164
	5-10-9	161.609	134.906	32.911	0.006763	0.004992	0.03226	1.716	0.155
0.4	5-10-11	185.612	151.300	32.992	0.007114	0.005497	0.04065	1.925	0.135
	5-10-12	192.457	152.292	34.248	0.006757	0.005610	0.04180	1.938	0.134
	5-10-15	186.921	147.143	33.400	0.006876	0.004870	0.03708	1.872	0.131
0.5	5-10-18	205.963	154.009	25.742	0.009216	0.006436	0.04951	1.959	0.130
	5-10-19	209.697	161.717	27.434	0.008934	0.006677	0.04974	2.057	0.134
	5-10-22	203.149	153.680	26.345	0.008899	0.006194	0.04729	1.955	0.131
0.6	5-10-24	239.397	176.500	26.274	0.009828	0.007018	0.05610	2.246	0.125
	5-10-25	233.485	173.070	26.473	0.009792	0.006475	0.05515	2.202	0.117
	5-10-29	228.339	167.729	25.910	0.009861	0.006527	0.05350	2.134	0.122

In Table 2, *P* is the impact pressure, $\dot{\varepsilon }$ is the average strain rate, $\sigma_{d}$ is the dynamic compressive strength, *E*_*d*_ is the dynamic elastic modulus, $\varepsilon_{d}$ is the peak strain, $\varepsilon_{0}$ is the strain when the sample deviates from the linear elastic segment, $\varepsilon_{max}$ is the limiting strain, *DIF* is the hardening factor and *K* is the softening factor. Here, *E*_*d*_ is the slope of an approximate straight line near 0.5 $\sigma_{d} \user2{ }$ in the stress–strain curve, and the hardening coefficient *DIF* and softening factor *K* are calculated from Eqs. (4) and (5)^26^.

4 $$DIF = \frac{{\sigma_{d} }}{{\sigma_{c} }}$$

5 $$K = \frac{{\varepsilon_{0} }}{{\varepsilon_{max} }}$$

## Dynamic characteristics analysis

### Characteristics of stress–strain curves

**Figure 4:**
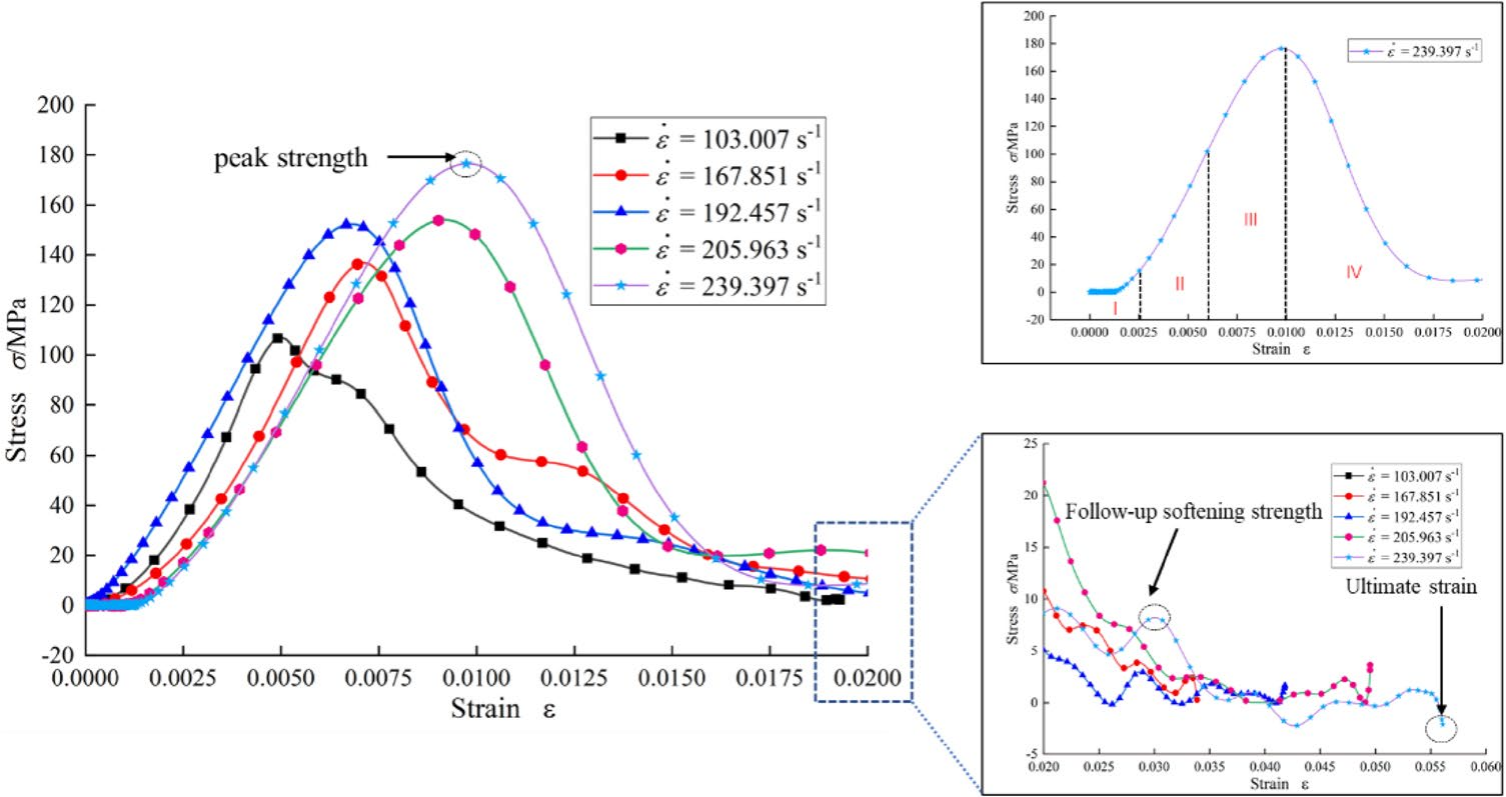
Typical stress–strain curves of graphite ore rock samples at different strain rates.

The stress–strain curves can not only reflect the mechanical properties of the rock materials, but also accurately describe the evolution characteristics of the rock in each stage during the loading process. Meanwhile, they can also explain the failure mechanism of the rock materials from the perspective of energy, which is the main means to study the mechanical behavior of the rock. Figure 4 shows the typical stress–strain curves of graphite ore rock samples.

According to the characteristics of the curves, the stress–strain curves can be divided into the compaction stage (I), the linear elastic stage (II), the crack evolution stage (III) and the unloading stage (IV). Where, the linear elastic stage refers to the stage in which the slope of the stress–strain curve remains basically unchanged, and the crack evolution stage refers to the curve stage between the strain value when the stress–strain curve deviates from the elastic stage and the peak strain value. Taking the stress–strain curve with the strain rate of 205.963 s^−1^ as an example, the stress–strain curve rapidly enters the linear elastic stage after a very short compaction stage, and then enters the crack evolution stage and unloading stage. Stress–strain curves at other strain rates can also be divided into the above four stages.

But under different strain rates, the evolution characteristics of the stress–strain curves are obviously different in every stage. Within the linear elastic stage of a curve, the slope decreases with the increase of strain rate to some degree, which indicates that the increase of strain rate weakens the anti-deformation ability of the graphite ore slightly. This conclusion is in line with the literature^37^. Meanwhile, from the perspective of peak strength and follow-up softening strength (the follow-up softening strength here refers to the bearing strength of the broken body after the sample is subjected to the impact load, which can be understood as the ability of the broken body to resist external load when the incident bar makes a secondary impact on the broken sample), with the increase of strain rate, the peak strength increases gradually, but the follow-up softening strength decreases gradually. Also, the ultimate strain that can be reached increases gradually with the increase of strain rate. This is an obvious post-peak softening phenomenon. The above phenomena indicate that the dynamic characteristics of the graphite ore rock have obvious strain rate effect, not only in the pre-peak stage, but also in the post-peak stage. In order to further explain these phenomena, the hardening coefficient (*DIF*) and the softening factor (*K*) will be used to analyze and discuss the dynamic characteristics of the graphite ore rock below.

### Hardening coefficient *DIF*

**Figure 5:**
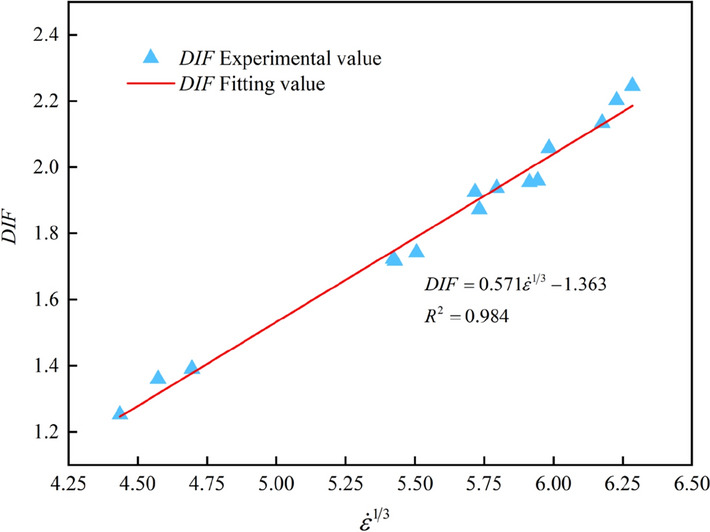
Hardening coefficient *DIF* changes with ${ }\dot{\varepsilon }^{{{1 \mathord{\left/ {\vphantom {1 3}} \right. \kern-0pt} 3}}}$.

For the strain rate effect of rock dynamic mechanical characteristics, Li et al.^8^ found that the dynamic compressive strength $\sigma_{d}$ is approximately proportional to the $\dot{\varepsilon }^{\frac{1}{3}}$ based on a large number of test data. Hao et al.^38^ proposed the use of hardening coefficient *DIF*(the ratio of dynamic strength to static strength) to further evaluate the variation rule of the dynamic strength of rock under different strain rates. According to the results of the two scholars, *DIF* and $\dot{\varepsilon }^{\frac{1}{3}}$ are fitted to obtain the functional relationship between them, as shown in Eq. (6). And the fitted curve is shown in Fig. 5.

6 $$DIF = 0.571\dot{\varepsilon }^{\frac{1}{3}} - 1.363\begin{array}{*{20}c} {} & {} \\ \end{array} \left( {R^{2} = 0.984} \right)$$

Equation (6) shows that the *DIF* still has a linear relationship with the $\dot{\varepsilon }^{\frac{1}{3}}$ with a high degree of fitting correlation coefficient *R*^2^ of 0.984. This perfectly demonstrates the variation of the *DIF* with the $\dot{\varepsilon }^{\frac{1}{3}}$. Equation (6) also illustrates that the *DIF* of the graphite ore rock samples has an obvious strain rate effect, which is the mechanical response of the rock material caused by the transformation from one-dimensional stress state to one-dimensional strain state^2^. That is, with the increase of strain rate, the number of cracks in the rock increases correspondingly, which needs a lot of energy to drive the crack extension and penetration. The loading time is so short that the rock does not have enough time to complete the accumulation, conversion, and release of energy. So, only by constantly improving the bearing capacity, can the rock resist the external load. However, under static load, the rock has a relatively long time to complete the accumulation, conversion and release of internal energy, and the static compressive strength of the rock only fluctuates around a certain stable value due to the influence of the inherent difference of rock. Therefore, the *DIF* increases with the increase of strain rate.

### Softening factor *K*

**Figure 6:**
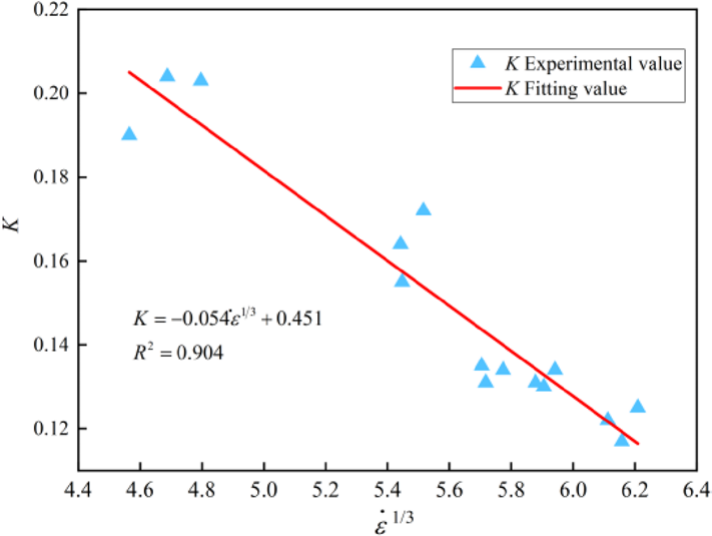
Softening factor *K* changes with ${ }\dot{\varepsilon }^{{{1 \mathord{\left/ {\vphantom {1 3}} \right. \kern-0pt} 3}}}$.

Studies have shown that rock materials soften to a certain extent with the increase of strain rate. Referring to the research conclusion of Li et al.^26^, under impact load, the relative proportion of rock elastic range in the whole strain range will gradually decreases with the increase of strain rate. Therefore, the ratio of elastic strain range to the overall strain range can be called the rock softening factor *K*. According to Eq. (5), *K* is between 0.117 and 0.204, as shown in Table 3. According to the variation characteristics of *K* with strain rate, the functional relationship between *K* and $\dot{\varepsilon }^{\frac{1}{3}}$ is obtained by curve fitting, as shown in Eq. (7). And the fitted curve is shown in Fig. 6.

7 $$K = - 0.054\dot{\varepsilon }^{\frac{1}{3}} + 0.451\begin{array}{*{20}c} {} & {} \\ \end{array} \left( {R^{2} = 0.904} \right)$$

**Table 3:** Rock sample crushing process.

$\dot{\varepsilon }$	Failure stage				
	Compaction	Crack initiation	Crack growth and transfixion	Fragments collision	Fragments falling
103.007 s^−1^					
167.851 s^−1^					
192.457 s^−1^					
205.963 s^−1^					
239.397 s^−1^

As can be seen from Eq. (7) and Fig. 6, with the increase of strain rate, the *K* gradually decreases. That is, the degree of softening gradually deepens. In the stress–strain curve shown in Fig. 3, the following softening strength gradually decreases and the ultimate strain gradually increases. A conclusion can be drawn that with the increase of strain rate, the relative values of elastic–plastic deformation and inhomogeneous plastic deformation of the ore samples gradually increase, and the crushing degree of the ore samples also deepens.

## Analysis of crushing characteristics

### High-speed photography of crushing process

High speed camera is one of the important tools used to observe the dynamic fracture process of rock samples. The equipment used in the high-speed photography experiment was the FASTCAM-SA1.1 High-speed Digital Camera produced by Photron Company. The camera has a high shooting speed and resolution, which can achieve a shooting speed of 5400 fps under the full frame resolution of 1024 × 1024, and shoot up to 675,000 fps at reduced resolution. The resolution of 320 × 320 was adopted in this test and the frame frequency was set as 10,000 fps. According to the image characteristics recorded by the high-speed photographic camera, the failure process of the samples can be divided into five stages, as shown in Table 3.

1. Compaction stage. The sample is just impacted, resulting in a short and small shrinkage deformation. The sample rapidly transfers from the microcrack compaction stage to the microcrack initiation stage, but no macroscopic cracks are formed at this stage.

2. Crack initiation stage. Shear cracks occur in the sample. The shear cracks start from the specimen near the bar end, then spread along the axial direction, and accompanied by a small number of secondary cracks.
3. Crack growth and transfixion stage. Secondary cracks continue to develop. Shear cracks extend along the axial direction, and begin to expand along the circumferential direction of the sample. At the same time, the shear cracks widen significantly, converging and transfixing with the secondary cracks, so that the sample is cut into fragments of different particle sizes. At this point, the incident bar begins to separate from the sample.
4. The collision stage of fragments. The incident bar and the sample are completely separated, and the fragments after impact are splashed to the end of the transmission bar at a certain initial velocity. Due to the different initial velocity of fragments, in this process, the fragments will collide and squeeze each other, and the sample is further broken.
5. The falling stage of fragments. The incident bar and transmission bar are completely separated from the sample. And the broken sample falls back under the action of gravity and accompanied by a small amount of rock powder.

When the incident bar and transmission bar contact again, the impact test is completed.

### Crushing mode and crushing form

**Figure 7:**
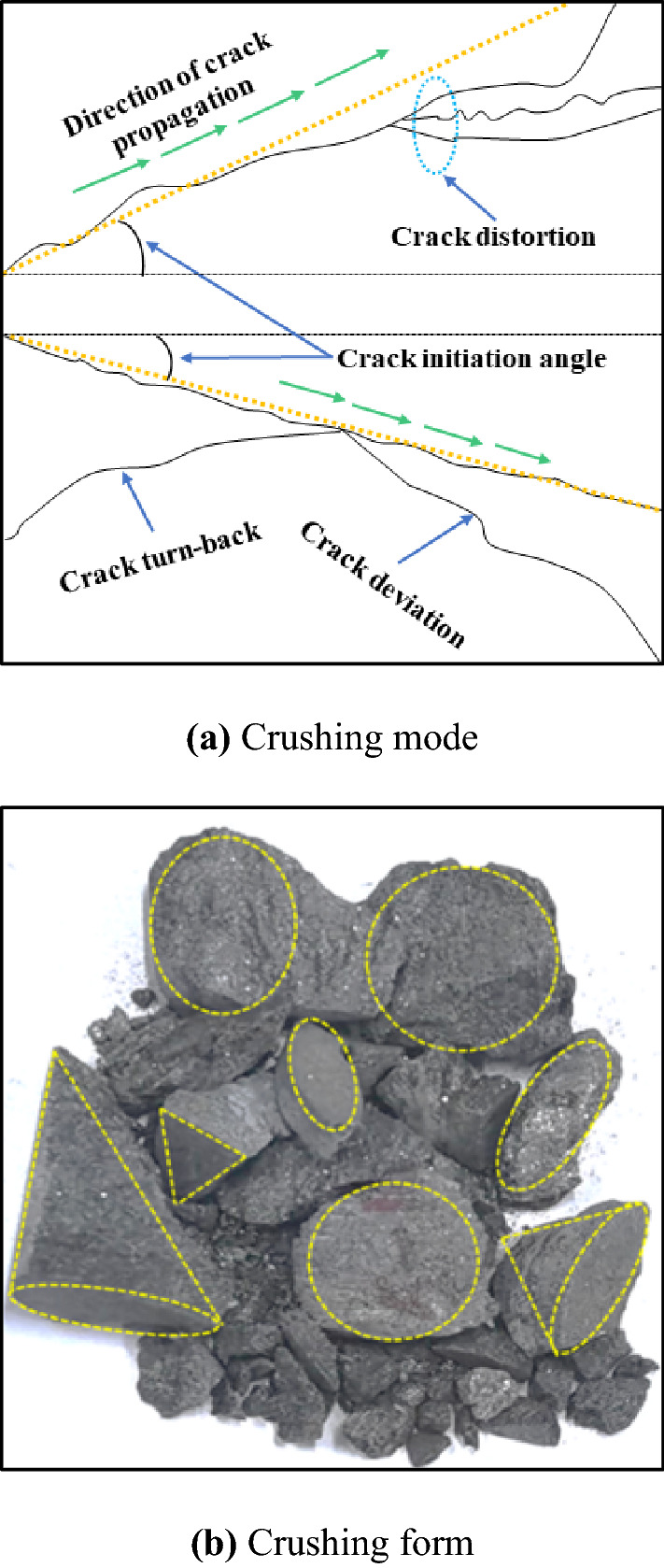
Schematic diagram of crushing mode and crushing form (**a**) crushing mode; (**b**) crushing form.

Figure 7a and b describe the crushing mode and crushing form of the samples. In terms of the crushing mode, the crushing of a sample is mainly shear failure, which is consistent with the conclusion in the literature^39^. Under the action of stress load, dilatancy deformation of a sample begins, followed by macroscopic cracks, which start from the part of a sample near the incident bar end and extend to the transmission bar end. The stress wave propagates back and forth between the incident bar, the sample and the transmission bar. The energy carried by the stress wave causes the cracks to expand, extend, converge and coalesce until the sample completely loses its bearing capacity. As the impact pressure increases, the strain rate increases, and the crack initiation velocity, the crack initiation angle and the crack number change obviously. In general, with the increase of strain rate, the crack initiation speed is accelerated, the crack initiation angle is gradually reduced, and the number of cracks is gradually increased. In terms of macroscopic crushing characteristics, the crushing size of samples decreases gradually.

In terms of the crushing form, the crushed fragmentations are mainly of triangular pyramid (or conical) shape, fine-grained and powdery, which is mainly related to the characteristics of the shear cracks. At the beginning of the crack, there is an angle between the shear crack and the axial direction. The crack extending direction does not significantly change until the lithology and the rock structure change or the initial crack converge or penetrate with other cracks. At this time, the crack extension direction shifts, twists or turns back, so that to form a crack network with triangle geometry. When the broken sample is cut into tripyramidal (or conical) fine granular fragments, the pulverized fragments will be formed due to the behaviors of cracks cracking, shear plane dislocation and collision of fragments, etc.

### Fragmentation distribution characteristics

**Figure 8:**
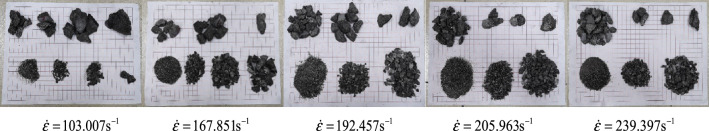
Sieving diagram of sample fragments at different strain rates.

Through the screening test, the proportion of fragmentation distribution in different particle size intervals was obtained. Figure 8 shows the sieving diagram of sample fragments at different strain rates. The cumulative mass percentage of each particle size on the screen was counted, and the average broken particle size and the fractal dimension were calculated, as listed in Table 4. And the fragmentation distribution character-istics of the graphite ore rock samples under different strain rates were analyzed.

**Table 4:** Fragmentation screening and fractal calculation.

*P*/MPa	Sample number	$\dot{\varepsilon }$	Cumulative mass percentage of each particle size on the screen/%								*d*_*s*_/mm	*D*_*a*_
			40–50 mm	31.5–40 mm	20–31.5 mm	16–20 mm	10–16 mm	5–10 mm	2.5–5 mm	< 2.5 mm		
0.2	5-10-3	103.007	28.239	36.052	13.453	12.919	2.134	3.659	1.791	1.753	31.655	1.654
	5-10-4	95.129	37.486	26.328	16.699	4.624	7.260	5.120	1.146	1.337	32.478	1.559
	5-10-5	110.328	28.360	19.514	15.604	9.301	17.008	5.125	2.733	2.354	27.894	1.728
0.3	5-10-7	167.851	13.946	21.465	18.192	10.908	14.842	10.947	4.168	5.532	23.276	2.003
	5-10-8	161.163	16.699	17.897	6.610	13.491	22.922	12.022	4.909	5.450	21.993	2.045
	5-10-9	161.609	12.880	17.147	11.842	8.035	25.029	14.033	5.344	5.690	20.782	2.017
0.4	5-10-11	185.612	0	27.903	5.519	13.760	24.492	14.258	6.478	7.589	18.214	2.056
	5-10-12	192.457	0	31.891	17.236	11.086	17.806	10.744	5.163	6.074	20.856	1.992
	5-10-15	186.921	0	19.299	18.451	2.735	27.157	17.951	6.125	8.282	17.070	2.072
0.5	5-10-18	205.963	0	6.303	11.524	12.606	30.046	21.462	8.082	9.977	13.300	2.134
	5-10-19	209.697	0	7.628	15.751	11.251	31.083	20.099	7.246	6.941	14.539	1.983
	5-10-22	203.149	0	6.238	12.437	17.745	27.431	17.474	8.834	9.841	13.818	2.156
0.6	5-10-24	239.397	0	0	13.010	12.525	26.505	26.424	10.263	11.273	11.460	2.083
	5-10-25	233.485	0	0	14.191	9.643	25.755	25.598	10.898	13.916	11.134	2.175
	5-10-29	228.339	0	0	7.772	12.197	33.359	25.394	10.081	11.197	10.898	2.063

**Figure 9:**
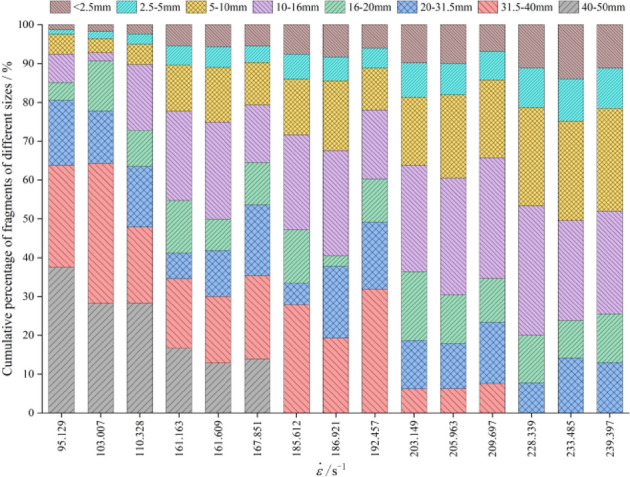
Cumulative percentage of fragments of different sizes.

As can be seen from Fig. 9, with the increase of the strain rate, the proportion of fragments with size between 31.5 and 50 mm gradually decreases, which of fragments with size less than 16 mm gradually increases. However, the proportion of fragments between 16 and 31.5 mm has no obvious change with the strain rate, indicating that strain rate has a more obvious effect on bulk and powder percentage. In order to quantitatively evaluate the effect of the strain rate on the size distribution of fragments, the average broken particle size (*ds*) and the fractal dimension (*D*_*a*_) were used for further analysis.

#### Average crushing particle size

The average broken particle size $d_{s}$ is defined to reflect the broken degree of the graphite ore rock, as shown in Eq. (8):

8 $$d_{s} = \frac{{\sum r_{i} d_{i} }}{{\sum r_{i} }}$$

where $d_{s}$ is the average broken particle size, $r_{i}$ is the mass percentage of fragments when the mesh diameter is $d_{i}$, and $d_{i}$ is the median of the mesh size grade. For example, $d_{i}$ = 45 mm when the mesh size grade is 40–50 mm.

**Figure 10:**
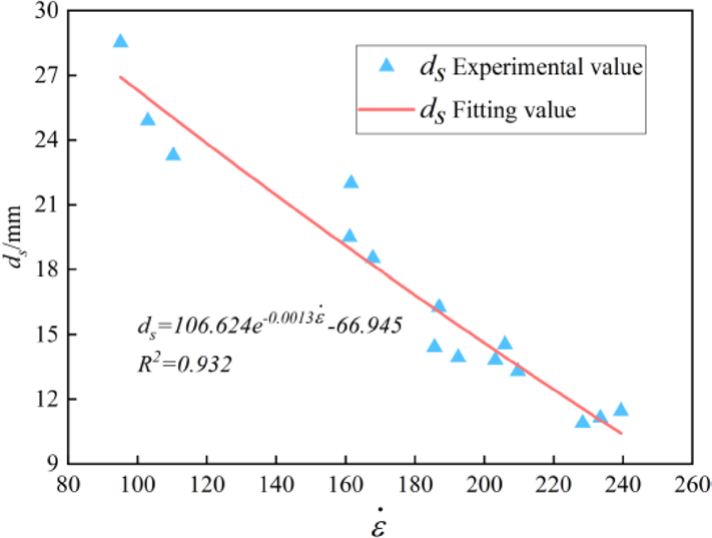
Relationship between average broken particle size with strain rate.

Power function, negative exponential function and linear function are used to fit the relationship between $d_{s}$ and $\dot{\varepsilon }$, respectively. It is found that there exists a good negative linear relationship between $d_{s}$ and $\dot{\varepsilon }$, as shown in Fig. 10. It indicates that a greater number of failure cracks activated by higher strain rate leads to more complete fracture degree of sample.

#### Fractal characteristics of crushing

**Figure 11:**
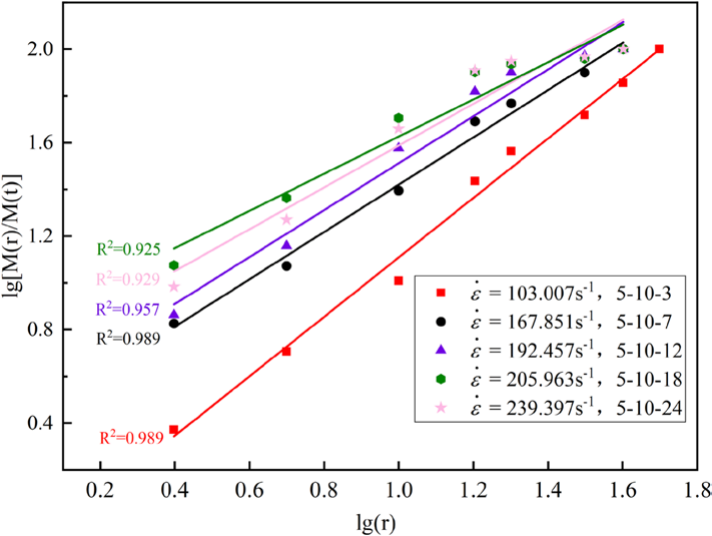
Total logarithmic cumulative grain size characteristic curve (part).

The mass-size distribution relation was used to calculate the fractal dimension *D*_*a*_, as shown in Eqs. (9) and (10).

9 $$D_{a} = 3 - \alpha$$

10 $$\alpha = \frac{{lg\left[ {M\left( r \right)/M\left( t \right)} \right]}}{lg\left( r \right)}$$

where $r$ is the standard sieve size, $M\left( r \right)$ is the cumulative mass of fragments with diameter less than $r$, *M(t*) is the total mass of sample fragments, $\alpha$ is the mass-size distribution index of the graphite ore rock broken fragments. Figure 11 shows a scatter plot in a cartesian coordinate system using $lg\left[ {M\left( r \right)/M\left( t \right)} \right]$ as the ordinate and $lg\left( r \right)$ as the abscissa. The slope of the obtained line is $\alpha$, and the calculation results of $\alpha$ and $D_{a}$ are listed in Table 5.

**Table 5:** Calculation results of fractal dimension.

Sample number	$\dot{\varepsilon }$	$\alpha$	*R*^*2*^	$D_{a}$
5-10-3	103.007	1.272	0.989	1.654
5-10-4	95.129	1.136	0.895	1.559
5-10-5	110.328	0.997	0.996	1.728
5-10-7	167.851	1.012	0.989	2.003
5-10-8	161.163	0.872	0.976	2.045
5-10-9	161.609	0.955	0.976	2.017
5-10-11	185.612	0.919	0.961	2.056
5-10-12	192.457	1.003	0.957	1.992
5-10-15	186.921	0.944	0.994	2.072
5-10-18	205.963	0.765	0.929	2.134
5-10-19	209.697	0.866	0.959	1.983
5-10-22	203.149	0.844	0.971	2.156
5-10-24	239.397	0.895	0.925	2.083
5-10-25	233.485	0.764	0.962	2.175
5-10-29	228.339	0.825	0.972	2.063

**Figure 12:**
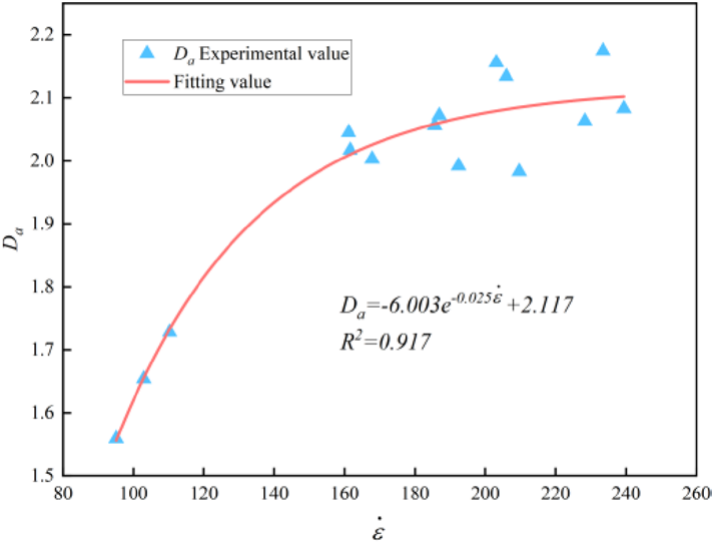
Fractal dimension curve with strain rate.

As shown in Table 5, *D*_*a*_ of the graphite ore rock samples under impact compression concentrates between 1.5 and 2.2, and within the strain rate range of 95–110 s^−1^, *D*_*a*_ is between 1.5 and 1.7, indicating that there are large scale broken fragments after the samples are crushed, and the crushing of the samples is not complete. At this time, the strain rate has an obvious influence on *D*_*a*_. When the strain rate is in the range of 160–240 s^−1^, *D*_*a*_ is between 1.9 and 2.2, indicating that the sample fragments are basically in a small scale interval and the crushing of the samples is relatively complete. At this time, the effect of strain rate on *D*_*a*_ is weakened. According to Table 6, the variation rule of fractal dimension of the graphite ore rock with strain rate is shown in Fig. 12, and the function relationship between them obtained by curve fitting is shown in Eq. (11).

11 $$D_{a} = - 6.003e^{{ - 0.025\dot{\varepsilon }}} + 2.117\begin{array}{*{20}c} {} & {} \\ \end{array} \left( {R^{2} = 0.917} \right)$$

**Table 6:** Distribution parameter calculation results.

${\dot{\varepsilon }}$	*m*	*F*_*0*_
103.007	1.902	186.328
95.129	1.641	180.004
110.328	1.798	194.156
167.851	2.056	232.365
161.163	2.186	224.912
161.609	1.997	231.308
185.612	2.278	247.607
192.457	2.390	244.908
186.921	2.246	242.01
205.963	2.315	250.576
209.697	2.405	259.472
203.149	2.368	247.984
239.397	2.628	274.128
233.485	2.475	274.779
228.339	2.376	270.309

As shown in Eq. (11) and Fig. 12, with the strain rate gradually increases, *D*_*a*_ gradually increases, indicating that the proportion of the broken fragments in the total mass of the large-scale samples gradually decreases. The degree of breakage of the samples becomes deeper and the fragmentation becomes smaller. But when the strain rate increases to a certain extent, *D*_*a*_ changes little. The influence of strain rate on the broken degree of the samples is not significant, which means that even the strain rate is increased at this time, the expected purpose of intensifying the breakage degree of the sample cannot be achieved. This is consistent with the phenomenon that increasing the charge or using high performance explosive has a limit effect on increasing the degree of rock fragmentation in practical blasting engineering. In conclusion, *D*_*a*_ can better reflect the crushing degree of samples at different strain rates, and has better guiding significance for determining the reasonable strain rate required by crushing rock mass.

## Dynamic damage constitutive model

### Definition of damage variables

There are a large number of randomly distributed defects in rock materials, which make the shape and strength of the rock micro-elements very different. Due to the large number of these micro-elements with different shapes and strengths, it is impossible to describe them one by one. Therefore, statistical methods can only be used to study them.

Assuming that the micro-element strength of rock follows Weibull distribution, its probability density function can be expressed as Eq. (12).

12 $$P\left( x \right) = \frac{m}{{F_{0} }}\left( {\frac{x}{{F_{0} }}} \right)^{m - 1} exp\left[ { - \left( {\frac{x}{{F_{0} }}} \right)^{m} } \right]$$

where $P\left( x \right)$ is the distribution function of rock micro-element strength, $x$ is the distribution variable of random distribution of rock micro-element strength, and, $m$ and $F_{0}$ are distribution parameters.

It is assumed that the rock is composed of a large number of micro-particles containing micro-cracks and other defects. The size is large enough in the spatial sense, but small enough in the mechanical level, and it can be regarded as a particle with the following properties: (1) The rock material is isotropic on the macro level, and the damaged body has isotropic characteristics; (2) The micro-element before failure shows linear elasticity, and the stress–strain relationship obeys Hooke's law; (3) the micro-element strength level *x* obey Weibull distribution, the probability density function *P*(*x*) as shown in Eq. (12).

The damage variable *D* is defined to reflect the damage degree of rock. The damage degree is related to the number of defects contained in the rock micro-element, which directly affects the strength of the rock micro-elements. Under a certain load, the statistical damage variable can be measured from the point of view of the number of micro-elements of failure, namely:

13 $$D = \frac{{N_{f} }}{N}$$

where, *D* is the statistical damage variable under load, *N*_*f*_ is the number of damaged micro-elements, and *N* is the total number of micro-elements.

Assuming that the strength at the time of failure of the rock micro-elements is *F*, and at any interval [*F*,*F* + *d*F], when external load increased from 0 to *F*, the Eq. (13) can be obtained.

14 $$N_{f} = N\mathop \smallint \nolimits_{0}^{F} \left( {\frac{m}{{F_{0} }}} \right)\left( {\frac{x}{{F_{0} }}} \right)^{m - 1} exp\left[ { - \left( {\frac{x}{{F_{0} }}} \right)^{m} } \right]dx$$

Further, Eq. (14) can be expressed as follows.

15 $$D = 1 - exp\left[ { - \left( {\frac{F}{{F_{0} }}} \right)^{m} } \right]$$

### Establishment of constitutive model

According to the strain equivalence hypothesis of Lemaitre^40^, the constitutive relation of rock damage can be established as follows.

16 $$\sigma = \sigma^{*} \left( {1 - D} \right) = E\varepsilon \left( {1 - D} \right)$$

where *σ* is the nominal stress, σ^*^ is effective stress, *E* is the elastic modulus, *ε* is the peak strain, and *D* is the damage variable.

According to D-P failure criterion, rock failure satisfies Eq. (17).

17 $${\text{F}} = \alpha_{0} I_{1} + \sqrt {J_{2} }$$

where $\alpha_{0} = \sin \varphi /\sqrt {9 + 3sin^{2} \varphi }$, *φ* is the internal friction angle of the rock, *I*_*1*_, *J*_*2*_ are the first invariant of the effective stress tensor and the second invariant of the effective stress offset respectively, and there are as follows.

18 $$I_{1} = \sigma_{1}^{*} + \sigma_{2}^{*} + \sigma_{3}^{*}$$

19 $$J_{2} = \frac{1}{6}\left[ {\left( {\sigma_{1}^{*} - \sigma_{2}^{*} } \right)^{2} + \left( {\sigma_{2}^{*} - \sigma_{3}^{*} } \right)^{2} + \left( {\sigma_{1}^{*} - \sigma_{3}^{*} } \right)^{2} } \right]$$

In conventional triaxial test of rock can be measured nominal stress *σ*_1_, *σ*_2_, *σ*_3_ (*σ*_2_ = *σ*_3_) and *ε*_1_, corresponding effective stress for *σ*_1_^*^, *σ*_2_^*^, *σ*_3_^*^ (*σ*_2_^*^ = *σ*_3_^*^). Rock elastic modulus and Poisson Ratio of *E* and *μ*, respectively. The following equations can be obtained from Hooke's law.

20 $$\varepsilon_{1} = \left( {\sigma_{1}^{*} - 2\mu \sigma_{3}^{*} } \right)/E$$

21 $$\sigma_{3}^{*} = \sigma_{2}^{*} = \sigma_{3} /\left( {1 - D} \right)$$

22 $$\sigma_{1}^{*} = \sigma_{1} /\left( {1 - D} \right) = E\varepsilon_{1} /\left( {1 - D} \right)$$

Further, the following equations can be obtained.

23 $$I_{1} = \frac{{\left( {\sigma_{1} + 2\sigma_{3} } \right)E\varepsilon_{1} }}{{\left( {\sigma_{1} - 2\mu \sigma_{3} } \right)}}$$

24 $$\sqrt {J_{2} } = \frac{{\left( {\sigma_{1} - \sigma_{3} } \right)E\varepsilon_{1} }}{{\sqrt 3 \left( {\sigma_{1} - 2\mu \sigma_{3} } \right)}}$$

In this test, the rock sample is under uniaxial impact compression, so $\sigma_{2} = \sigma_{3} = 0$ and $\varepsilon_{1} = \varepsilon$. It can be seen from Table 1 that $\varphi = 29.50^\circ$, so Eq. (17) can be written as follows.

25 $$F = \left( {\frac{\sin \varphi }{{\sqrt {9 + 3sin^{2} \varphi } }} + \frac{1}{\sqrt 3 }} \right)E\varepsilon = 0.735E\varepsilon$$

Combining Eqs. (15), (16) and (25), the constitutive relation of rock micro-element strength subject to Weibull distribution can be obtained, shown as Eq. (26).

26 $$\sigma = E\varepsilon exp\left[ { - \left( {\frac{0.735E\varepsilon }{{F_{0} }}} \right)^{m} } \right]$$

### Parameter calculation and constitutive model modification

According to Eq. (26), the rock damage constitutive model can be obtained after determining *m* and *F*_*0*_. In the uniaxial impact compression test, *m* and *F*_*0*_ can be determined by the peak strength point $C\left( {\varepsilon_{d} ,\sigma_{d} } \right)$ and the elastic modulus *E*_*d*_ of the stress–strain curves. The slope at the peak strength point $C\left( {\varepsilon_{d} ,\sigma_{d} } \right)$ is 0, so the Eq. (27) can be obtained.

27 $$\left. {\frac{d\sigma }{{d\varepsilon }}} \right|_{{\varepsilon = \varepsilon_{d} }} = E\left\{ {\left[ {1 - m\left( {\frac{0.735E\varepsilon }{{F_{0} }}} \right)^{m} } \right]exp\left[ { - \left( {\frac{0.735E\varepsilon }{{F_{0} }}} \right)^{m} } \right]} \right\} = 0$$

Meanwhile, $\sigma_{d}$ at peak point $C\left( {\varepsilon_{d} ,\sigma_{d} } \right)$ satisfies the relation of Eq. (28).

28 $$\sigma_{d} = E_{d} \varepsilon_{d} exp\left[ { - \left( {\frac{{0.735E_{d} \varepsilon_{d} }}{{F_{0} }}} \right)^{m} } \right]$$

From Eq. (27) and Eqs. (28), (29) and Eq. (30) can be obtained as follows.

29 $$m = \left[ {1/\ln \left( {\frac{{E_{d} \varepsilon_{d} }}{{\sigma_{d} }}} \right)} \right]$$

30 $$F_{0} = 0.735E_{d} \varepsilon_{d} m^{{\left( \frac{1}{m} \right)}}$$

**Figure 13:**
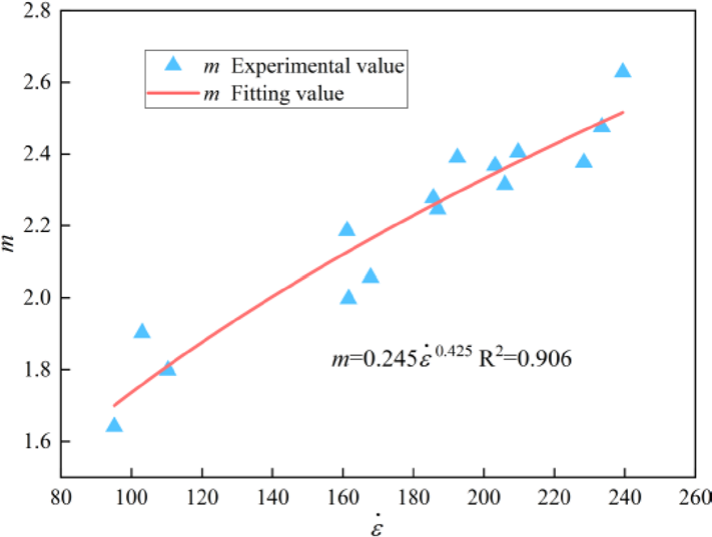
Fitting relationship between *m* and strain rate.

**Figure 14:**
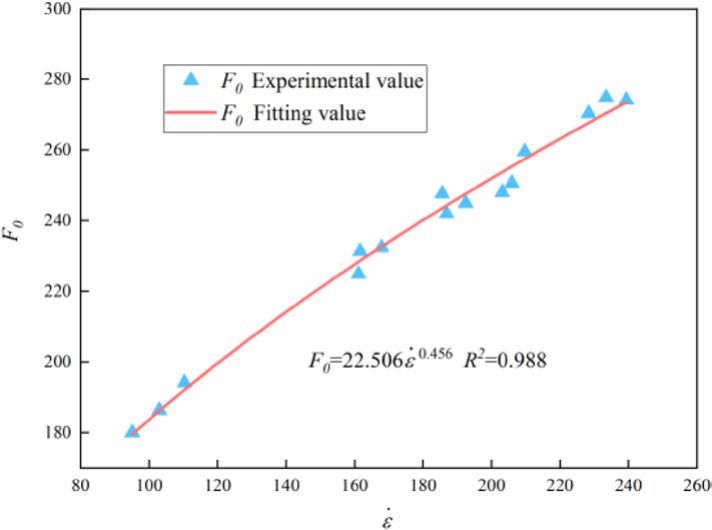
Fitting relationship between *F*_*0*_ and strain rate.

Substituting the data in Table 3 into Eqs. (29) and (30), the calculation results of parameters *m* and *F*_*0*_ are listed in Table 6. It can be found that distribution parameters (*m* and *F*_*0*_) are significantly correlated with strain rate. Scatter plots were drawn with *m* and *F*_*0*_ as ordinate and strain rate as abscissa and non-linear fitting was carried out, as shown in Figs. 13 and 14. Fitting relations of *m* and *F*_*0*_ with strain rate were obtained as shown in Eqs. (31) and (32).

31 $$m = 0.245{\dot{\varepsilon }}^{0.425}\,\,\,R^{2} = 0.906$$

32 $$F_{0} = 22.506{\dot{\varepsilon }}^{0.456}\,\,\,R^{2} = 0.988$$

Substituting Eqs. (31) and (32) into Eq. (26), the modified dynamic damage constitutive model of graphite ore rock can be obtained, as shown in Eq. (33).

33 $$\sigma = E\varepsilon exp\left[ { - \left( {\frac{0.735E\varepsilon }{{22.506{\dot{\varepsilon }}^{0.456} }}} \right)^{{0.245{\dot{\varepsilon }}^{0.425} }} } \right]$$

### Model verification

**Figure 15:**
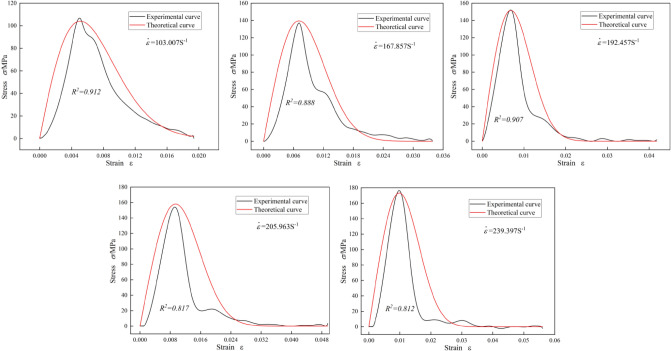
Theoretical curves and experimental curves of graphite ore rock under different strain rates.

The modified dynamic damage constitutive model was used to calculate the theoretical stress–strain curves of the graphite ore at different strain rates, which were compared with the experimental curves, as shown in Fig. 15. Comparing the constitutive model curve of the graphite ore rock with the test curve at different strain rates, it can be seen that the constitutive model curve established in this paper has a relatively good consistency with the test curve (correlation coefficient R^2^ > 0.81). After reasonably modifying the constitutive model by establishing the correlation between Weibull distribution parameters (*m* and *F*_*0*_) and the strain rates, the strain rate effect of peak stress, peak strain and dynamic elastic modulus of the graphite ore rock can be well reflected by the model curve. Although there are some local deviations between the model curve and the experimental curve, the curve boundary characteristics such as peak strain and peak stress are in good agreement with the experimental results, which indicates the rationality of the model.

## Discussions

The dynamic mechanical properties, crushing characteristics and energy consumption characteristics of rock materials can be studied by SHPB test, and more and more achievements have been produced at present, but there are still some problems to be solved. For example, during the test, there are certain differences in strain rates under the same impact pressure due to the optional adjustment gradient is large when the impact pressure is adjusted. Adjusting the impact pressure with a gradient of 0.1 MPa will inevitably cause unavoidable errors. Therefore, it is necessary to research and improve more accurate, adjustable gradient of the voltage regulator.

In addition, in the study of rock fragment distribution characteristics, firstly, the square hole sieve with different size are used to grade for screen, secondly, the electronic balance is used to weigh, and finally statistical calculation of its fragment distribution, which is very troublesome, time consuming and energy consuming. Therefore, an automatic screening and calculation of the rock fragment distribution device will greatly reduce the complexity of the test.

In the process of establishing the statistical strength damage constitutive model of graphite ore rock, some idealized assumptions are made, while the mechanical behavior of actual rock materials is uncertain, so there is a certain deviation between the model curve and the test curve. In the subsequent research, the damage constitutive model with better effect in accordance with the experimental results can be explored from the aspects of combining the statistical damage theory and component combination theory or changing the damage distribution function.

## Conclusions

In this paper, the SHPB tests of graphite ore rock at different strain rates were carried out, combining with high-speed photography and screening tests, the dynamic characteristics and dynamic crushing characteristics of graphite ore rock at different strain rates were analyzed, and the strength type dynamic damage constitutive model of graphite ore rock was established. The conclusions are as follows.

1. The impact test results show that the strain rate effect of the dynamic characteristics of the graphite ore rock is not only in the pre-peak stage, but also in the post-peak stage. Specifically, the hardening coefficient (*DIF*) has a positive linear correlation with ($\dot{\varepsilon }^{{{1 \mathord{\left/ {\vphantom {1 3}} \right. \kern-0pt} 3}}}$), while the softening factor (*K*) has a negative linear correlation with ($\dot{\varepsilon }^{{{1 \mathord{\left/ {\vphantom {1 3}} \right. \kern-0pt} 3}}}$).
2. High-speed photographic tests show that the dynamic crushing process of the graphite ore rock can be divided into five stages, which are compaction, crack initiation, crack development and coalescence, fragmentation collision and fragmentation fall.
3. The failure mode of the graphite ore rock under impact load is mainly shear failure, and the broken blocks are mainly tripyramidal (or conical) fine granular and powdery.
4. Sieve tests show that the broken fragments of the graphite ore rock accord with the fractal geometric features. The average broken particle size (*d*_*S*_) decreases linearly with the increase of the strain rate, and the fractal dimension (*D*_*a*_) increases as weak exponential function with the increase of the strain rate.
5. The strain rate effect of the dynamic characteristics such as peak stress, peak strain and dynamic elastic modulus of the graphite ore rock can be well reflected by the model curve, which proves the rationality of the dynamic damage constitutive model established in this paper.
6. A more accurate constitutive model can be explored by combining statistical damage theory and component combination theory or changing the distribution function of damage variables.

## Data Availability

The datasets used and analyzed during the current study available from the corresponding author on reasonable request.
